# Kinematic and biomimetic assessment of a hydraulic ankle/foot in level ground and camber walking

**DOI:** 10.1371/journal.pone.0180836

**Published:** 2017-07-13

**Authors:** Xuefei Bai, David Ewins, Andrew D. Crocombe, Wei Xu

**Affiliations:** 1 Department of Mechanical Engineering Sciences, University of Surrey, Guildford, United Kingdom; 2 Gait Laboratory, Queen Mary’s Hospital, Roehampton, London, United Kingdom; Northwestern University, UNITED STATES

## Abstract

Improved walking comfort has been linked with better bio-mimicking of the prosthetic ankle. This study investigated if a hydraulic ankle/foot can provide enough motion in both the sagittal and frontal planes during level and camber walking and if the hydraulic ankle/foot better mimics the biological ankle moment pattern compared with a fixed ankle/foot device. Five active male unilateral trans-femoral amputees performed level ground walking at normal and fast speeds and 2.5° camber walking in both directions using their own prostheses fitted with an “Echelon” hydraulic ankle/foot and an “Esprit” fixed ankle/foot. Ankle angles and the Trend Symmetry Index of the ankle moments were compared between prostheses and walking conditions. Significant differences between prostheses were found in the stance plantarflexion and dorsiflexion peaks with a greater range of motion being reached with the Echelon foot. The Echelon foot also showed significantly improved bio-mimicry of the ankle resistance moment in all walking conditions, either compared with the intact side of the same subject or with the “normal” mean curve from non-amputees. During camber walking, both types of ankle/foot devices showed similar changes in the frontal plane ankle angles. Results from a questionnaire showed the subjects were more satisfied with Echelon foot.

## Introduction

Gait analysis is commonly used in the assessment of different prosthetic components to support the design, optimisation and selection of prostheses. Recently, there has been a series of gait studies on the functional performance of a clinically available ankle-foot hydraulic prosthetic device [1–5]. The hydraulic ankle-foot device is a passive single axis articulating design that allows custom control of the resisting moment. The gait from amputees with a hydraulic prosthetic ankle was compared with that using a fixed device and it was found that the hydraulic ankle/foot enabled decreased residual limb internal stresses, increased subjects’ self-selected walking speed, smoother and more rapid progression of prosthetic side plantar centre of pressure, and increased hip and knee joint power in both limbs [1–4].

Most of the previous tests with hydraulic prosthetic ankles/feet were only carried out on level ground walking conditions [1–3], with only one study including outdoor walking on a slope [4] and another one study involved indoor descending slope [5]. Adaption to uneven ground, however, is an important function of an ankle/foot system for daily living. Wirta et al. first applied camber walking (where the slope is inclined laterally to the walking direction, also known as cross-slope or side-slope) in the assessment of six different types of prosthetic ankle-foot devices and the lack of side to side motion was reported to be a drawback of conventional single axis prosthetic ankle-foot devices [6]. More recently, research on camber walking has been carried out to evaluate a newly developed prosthetic ankle-foot system [7], to investigate the differences between physiological ankles and (unspecified) prosthetic ones [8, 9], and to study the gait strategies of amputees in stability-challenging environments [10–12]. It has been found by Villa et al. that the (unspecified) prosthetic ankles/feet showed reduced inversion/eversion ankle angle on camber walking compared with non-amputees due to the lack of mobility in the frontal plane [8, 9]. Therefore tests on camber surface are needed in the assessment of hydraulic ankles/feet. For the hydraulic foot and the other feet used for comparison purpose in the previous studies, it would be expected that some inversion/eversion would be achieved by the deformation of the toe and heel springs [1–4]. No difference would necessarily be expected in this (frontal plane) movement as the structure of the other feet is the same and the hydraulic ankle only permits motion in the sagittal plane. However, the use of the hydraulic ankle may change the gait/posture of the subject and cause increased or reduced requirements in side-to-side motion. In addition, previous studies have shown the rate of centre of mass and plantar centre of pressure transfer (‘roll-over characteristics’) is altered with addition of the hydraulic ankle [2, 3], which may affect the spring deformation and lead to subtle changes in inversion/eversion.

The mimicking of human ankle biomechanics is emphasised in the design of prosthetic ankle-foot systems to enable efficient and comfortable walking of amputees [13, 14]. The shape of the prosthetic ankle moment waveform has been linked to the walking experience of amputees using a mathematical model [15, 16]. A substantial decrease in stresses on the residual limb was found when the resisting moment at the prosthetic ankles conformed more closely to the biological moment [16, 17]. Therefore, the similarity between the prosthetic ankle and the biological ankle moment waveforms might be used to assess and compare the performance of different prosthetic ankle-foot systems. Although many parameters that are relevant to prosthetic ankle moments have been considered in the investigations with lower limb amputees [18], so far, there is no published literature that reports the shape of the prosthetic ankle moment waveform as a parameter in the assessment of prosthetic ankle/foot devices.

In previous gait studies, three methods have been *most* commonly used in the comparison of kinematic and kinetic time curves: Coefficient of Multiple Correlation (CMC), Principal Component Analysis (PCA), and Trend Symmetry Index (TSI). CMC is a quantitative method that uses the standard deviation to mean values from a group of waveforms. This method was initially introduced in the assessment of kinematic marker placement repeatability [19]. CMC is sensitive to the differences in shift or multiplier of magnitude between kinematic or kinetic waveforms. This is commonly caused by marker displacement in motion capture systems or errors in segment parameters when using an inverse dynamics approach [20]. PCA has been applied in the research that investigates gait symmetry in able-bodied subjects and is mostly used with large sample sizes [21]. TSI is a more recently introduced method that quantifies the similarity between two waveforms using an eigenvector [22]. The TSI value is not affected by either shift or magnitude differences in two waveforms and provides quantitative results for each individual [22]. So far, TSI has been used in studies that compare the running gait of able-bodied subjects and in assessing different prosthetic knees [23, 24]. Other methods that has been used to identify the gait asymmetry from the time curves of biomechanical variables include cross-correlation [25, 26] and region of deviation analysis [27, 28],

The primary aim of this study is to evaluate the function of a hydraulic ankle-foot device on level and camber surface walking by comparison with a fixed ankle-foot device. The evaluation has two aspects, a) the kinematic assessment of the prosthetic foot sagittal plane motion and b) the biomimetic assessment of the prosthetic ankle moment to investigate whether the hydraulic prosthetic device could provide a resistance moment that has better bio-fidelity than fixed prosthetic ankle/foot devices. The secondary aim of this study is an assessment of the inversion/eversion provided by each prosthetic ankle-foot device.

## Methods

### Subjects

Five male trans-femoral amputee (TFA) subjects, who use their prostheses on a daily basis, participated in this study. Information of the subjects is given in Table 1. The length of the residual limb was measured from the anterior superior iliac spine to the distal end of the stump. A further 12 non-amputee (NA) subjects (5 male and 7 female, age: 26±2years, weight: 68±15kg, height: 1.71±0.07m) participated in this study to demonstrate the normal ranges of relevant parameters and provide a mean biological ankle moment curve for computing normalcy TSI. The ethical approval of this study was granted from the National Research Ethics Service Committee London—Bloomsbury and consents were obtained from all participants prior to the data collection.

**Table 1:** Individual details and the prostheses of the five subjects.

ID	Prosthetic side	Years of using prostheses	Prosthetic knee^a^	Prosthetic feet^a^	Age (years)	Weight (kg)^b^	Height (m)	Stump length (m)
TF1	R	13	KX06	EchelonVT	27	81	1.81	0.55
TF2	L	22	KX06	Elan	65	115	1.82	0.38
TF3	R	4	Linx	Linx	36	113	1.85	0.42
TF4	R	28	smart IP	Elan	57	123	1.84	0.42
TF5	L	5	Linx	Linx	27	105	1.84	0.47

^a^ The brand of all prosthetic knees and feet are Endolite.

^b^ Includes the weight of prostheses with the Esprit foot

### Prosthetic feet

Two different types of prosthetic feet were tested. One type of prosthetic foot, the Echelon (Blatchford & Sons Ltd., Basingstoke, UK), contains a hydraulic single axis articular joint that is adjustable in the dorsiflexion and plantarflexion range of motion and in the resistance moment, while the other type of the prosthetic foot, the Esprit (Blatchford & Sons Ltd., Basingstoke, UK), does not contain an articular ankle joint. The structures below the “ankle” joint are the same in both models, and include carbon toe and heel springs. For the feet selected it would be expected that some inversion/eversion would be achieved by the deformation of the toe and heel springs.

### Data collection protocol

The design of the protocol is based on the method introduced by van der Linden [29]. The subject was asked to change into shorts and the shoes that they normally use. The prosthetic device (Esprit or Echelon) that differed most from each subject’s normal prosthetic ankle/foot was the first to be attached to their prosthetic limb by the prosthetist. This was done to maximise familiarisation time, as in addition to dedicated practice time with the prosthetic limb, subjects also stood/walked during anthropometric measurement and marker placement. The prosthetic segment parameters required for biomechanical modelling were measured and recorded at the same time using the method described by Goldberg [30]. The optimisation of alignment and adjustment of the prostheses was agreed by both the prosthetist and the subject for the best comfortable experience. No change was made to the other prosthetic components except for changes in the shank tube length to enable use of the different prosthetic ankle-foot devices. For subjects TF3 and TF5 who used a Linx system, the prosthetist altered the limb setup so that the knee and ankle operated independently as conventional Orion and Echelon devices respectively. Each subject was given time to practise walking in the laboratory until they felt confident to start the data collection. Anthropometric parameters were then measured with the first prosthetic ankle/foot fixed as well as the parameters of the residual limb.

A modified Helen Hayes marker set that contains eleven short base markers and 4 wand markers was attached to the subject. The short base markers were placed on the intact side as follows: sacrum (between the left and right posterior superior iliac spines), ASIS (over the anterior superior iliac spines), knee (laterally on the knee joint line), ankle (on the lateral malleolus), toes (on the 1st metatarsal head and on the 5th metatarsal head). On the prosthetic side, the knee marker and ankle marker were placed laterally to the centre of rotation of the prosthetic joint when the prostheses contain mechanical articular structures. The ankle marker on Esprit foot was placed laterally to the centre of the distal end of the shank tube. The toe markers on both prosthetic feet were located at the same position as on the intact side. The wand markers were placed as follows: laterally on the thigh (approximately midway on a line between the greater trochanter and the knee) and laterally on the shank (approximately midway on a line between the knee and the lateral malleolus). The subject was asked to stand with the medial foot contacting two sides of a customised foot template that had been previously used in another study [31]. The thigh wand marker was then aligned with the greater trochanter and knee marker, and the shank wand marker was aligned with the knee marker and lateral malleolus.

The kinematic data was recorded at 120 Hz using an 11-camera motion capture system (ProReflex, Qualisys AB, Sweden) and the ground reaction forces (GRFs) were collected at 240 Hz from two force platforms (FP), (AMTI, USA, MODEL: BP400600HF-2000). The level walkway was 8 m in length. A mobile camber (6 m length, 1 m width and 2.5° incline) that was compatible with AMTI force plates was built according to Simon’s design concept [32].

**Fig 1:**
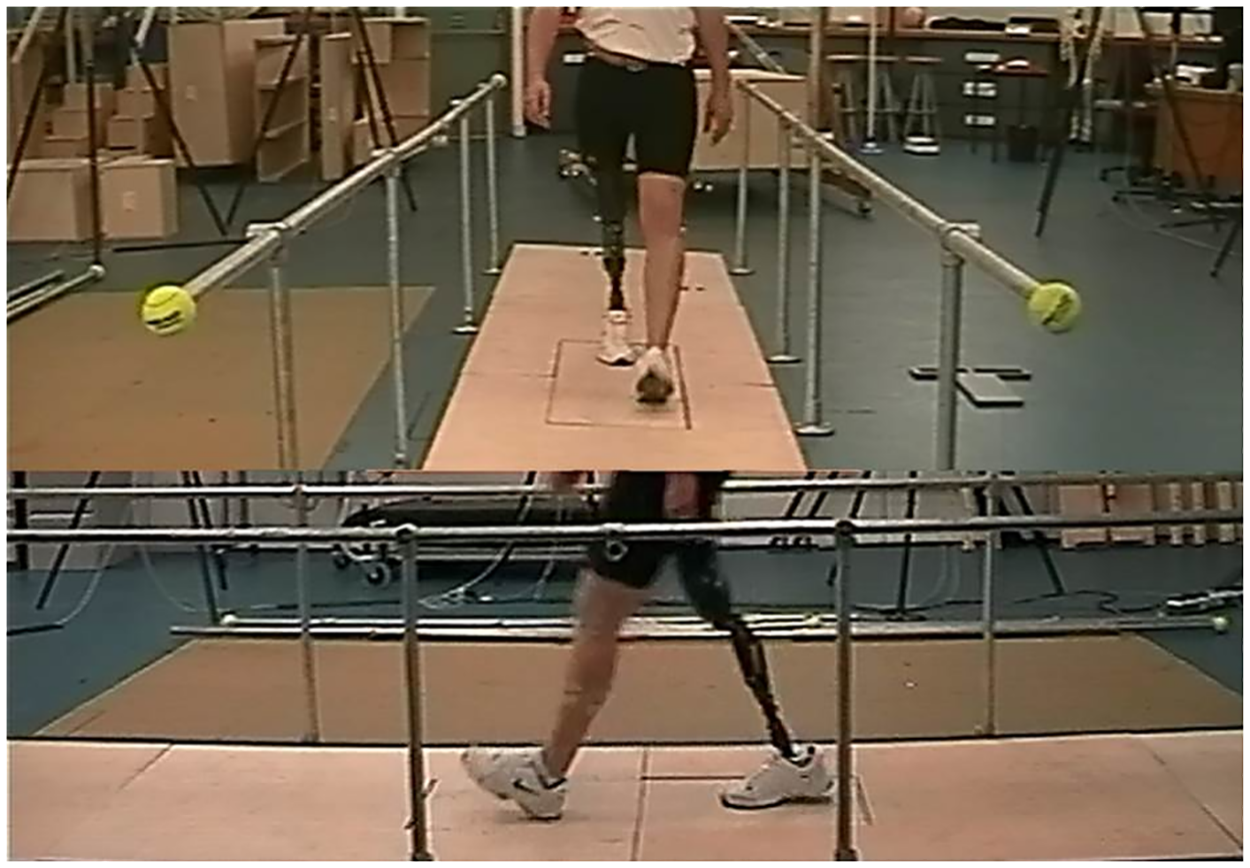
One trans-femoral amputee subject walking on the 2.5° camber walkway.

Each subject was then asked to perform (1) level ground self-selected normal speed walking, (2) level ground self-selected fast speed walking, (3) camber walking with prosthetic side higher, and (4) camber walking with intact side higher. Subjects were given time to practise until they could make clean single foot contact with each force plate before each activity was recorded and were encouraged to rest between the different walking condition tests. Five successful trials (containing whole gait cycles with complete kinematic data and ‘clean’ single foot contact with each force plate) in each walking condition were recorded. Fig 1 demonstrates a subject walking on the 2.5° camber. A short questionnaire abridged from a previous study on prosthetic ankle units was answered by the subjects after finishing each set of walking tests to give subjective assessment of the prosthetic ankles/feet [33]. The questionnaire was used to check a) if there was a conflict between prosthetic users’ walking experience and objective evaluation results (especially considering this is the first time that symmetry and normalcy TSI of ankle moment have been used in the assessment of prosthetic feet); and b) if there were any issues with the prosthetic feet that could not be observed from the parameters being measured. The prosthetist then attached and adjusted the second prosthetic foot for the subject and the test programme was repeated.

### Data processing

The markers were labelled in Qualisys Track Manager (Qualisys, Sweden, version 2.6.682) and exported into Visual3D (C-Motion, USA, Student Edition, version 5.00.16) for biomechanical modelling and future computing of relevant parameters. Gaps in the kinematic data of no more than 10 frames were filled and the data were then low pass filtered at 6 Hz (zero lag, 4th order, Butterworth filter). Biomechanical models were then generated for each individual with each type of prosthetic foot according to the measured anthropometric and prosthetic segment data. For a prosthetic ankle-foot device, the “ankle” motion is contributed to by the deformation of foot structures, or rotation of the articular ankle joint, or a combination of both. The relative position between the foot segment and shank segment therefore represents the total change of “ankle” angle achieved by the whole ankle-foot system in all directions. Full details of the modelling approach, including joint centre and (local) limb segment definitions and joint angle calculations, are given in Collins et al. [31]. However, to summarise, in this study, the (local) frontal plane of the shank segment is defined using the knee joint marker, shank wand and the ankle joint marker, and the (local) frontal plane of the foot segment is defined using the ankle marker and the 1^st^ and 5^th^ metatarsal head markers. In essence movement between these two frontal planes is used to define ankle inversion/eversion. In order to provide a systematic assessment for different feet and ground conditions, the analysis considered all the key variables from this data collection protocol. Four of the most commonly reported spatial–temporal parameters in lower limb amputees’ gait, walking speed, stride length, step length (on both sides sides), and stance time (on both sides, as a percentage of a gait cycle) were computed [18]. The ankle dorsiflexion/plantarflexion and inversion/eversion angles were calculated and normalised by the standing posture from the foot template. The peak plantarflexion, peak dorsiflexion and mid-stance (at 50% of stance phase) eversion angles during stance phase were then extracted. The sagittal plane ankle moment was computed using an inverse dynamics approach and time-normalised to 101 values over the stance phase to calculate symmetry TSI and normalcy TSI.

TSI quantifies the similarity between two waveforms that contain the same number of elements (values). The calculation of the TSI is based on the steps given by Fellin et al. [23] and the simplification inspired by Kaufman et.al [24]. In summary, the key steps are:

1. The elements of two waveforms were imported into one matrix with each pair of elements at the same time point i as a row. In this study, this generates a matrix with 101 time point (rows) and 2 time-normalised ankle moment values (columns).
2. The mean value of each curve was subtracted from each individual time point on the curve:
  $$\left\{\begin{array}{c} X_{Ti}\\ Y_{Ti} \end{array}\right\}=\left\{\begin{array}{c} X_{i}\\ Y_{i} \end{array}\right\}-\left\{\begin{array}{c} X_{m}\\ Y_{m} \end{array}\right\}$$ (1)
  where *X*_*i*_ and *Y*_*i*_ are the original time points from the right and left waveform respectively, and *X*_*m*_ and *Y*_*m*_ are the average values of each waveform. *T*_*i*_ represents the translated elements.
3. Singular value decomposition was then applied to the matrix to obtain the singular values.
4. The square of the second singular value is divided by the square of the first singular value.
5. The number obtained in step 4 was subtracted from one.

A TSI value ranges from 0 to 1. A value of 1 represents perfect trend similarity between the two waveforms and a lower value indicates less similarity. The symmetry TSI was computed by importing the time curves from the prosthetic and intact sides of the same subject into the matrix. Because all of the NA subjects were right leg dominated and all the amputee subjects stated they were intact leg dominated, the normalcy TSI was computed by comparing the prosthetic side of each amputee subject (or left side of each non-amputee subject) to the mean curve from the left sides of the entire NA group.

### Statistical analyses

Statistical analysis was applied to the spatial–temporal parameters, extracted values in ankle kinematics, and two types of TSI values in the amputee group only. A repeated measures two-way ANOVA was performed with walking conditions (activity, containing 4 levels: level normal speed, level fast speed, camber prosthetic side higher, and camber intact side higher) and models of prosthetic ankle/feet (foot, containing 2 levels: Echelon and Esprit) as two within subject factors. Post-hoc analyses were conducted with post-hoc Tukey tests. Statistical analysis was performed in IBM SPSS (IBM, USA, version 22.0.0.0) and the level of significance was set at p = 0.05.

## Results

One subject (subject TF4) fell during a trial of level ground normal speed walking with the first fitted prosthetic ankle/foot. The subject was not injured but for safety consideration, he was not asked to perform fast speed walking with either type of prosthetic ankle/foot. Another subject (subject TF2) had difficulties in getting clean single foot contacts with both force plates due to the large stride width in all tested walking conditions. Although the subject could deliberately reduce stride width to enable clean single foot contacts on both sides, as a “common” and “comfortable” walking condition was expected, we focused on clean single foot contacts from the prosthetic side only. Therefore the symmetry TSI of subject TF2 has not been calculated.

### Spatial–temporal parameters

**Fig 2:**
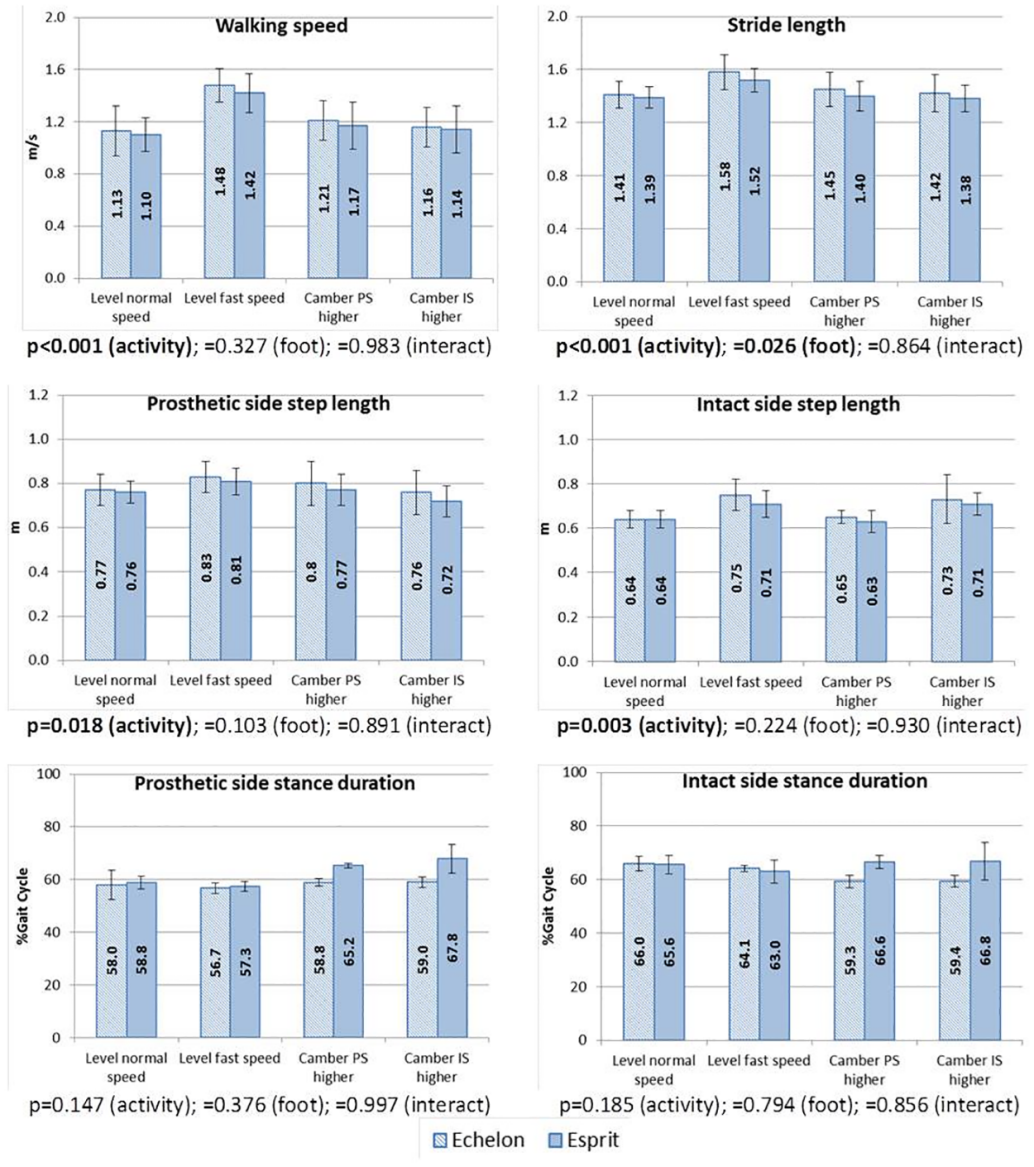
Mean values and standard deviations of the spatial–temporal parameters. The p-values of the repeated measures two-way ANOVA are given below each chart. PS = prosthetic side, IS = intact side.

The results of walking speed, stride length, step length, and stance time in all walking conditions, including p values of the ANOVA, are given in Fig 2. As showed in Fig 2, the significant differences in spatial-temporal parameters were found between different walking conditions (activity) and the only difference between prostheses (foot) is that when using the Echelon foot, the stride length was greater than when using the Esprit foot (p = 0.026). According to the post-hoc test results, the level ground fast walking speed and stride length are significantly greater than for all other walking conditions (p<0.001 in all relevant post-hoc results), while camber walking speed showed no significant difference compared with level ground normal walking speed. The fast walking also showed greater step length than level normal speed walking (prosthetic side: p = 0.028; intact side: p = 0.003), camber walking with prosthetic side higher (intact side: p = 0.005), and camber walking with the intact side higher (prosthetics side: p = 0.002). Besides, when walking on the camber with the intact side higher, the step length at the intact side was greater than both level normal speed walking (p = 0.006) and camber walking from the other direction (p = 0.010).

### Ankle kinematics

**Fig 3:**
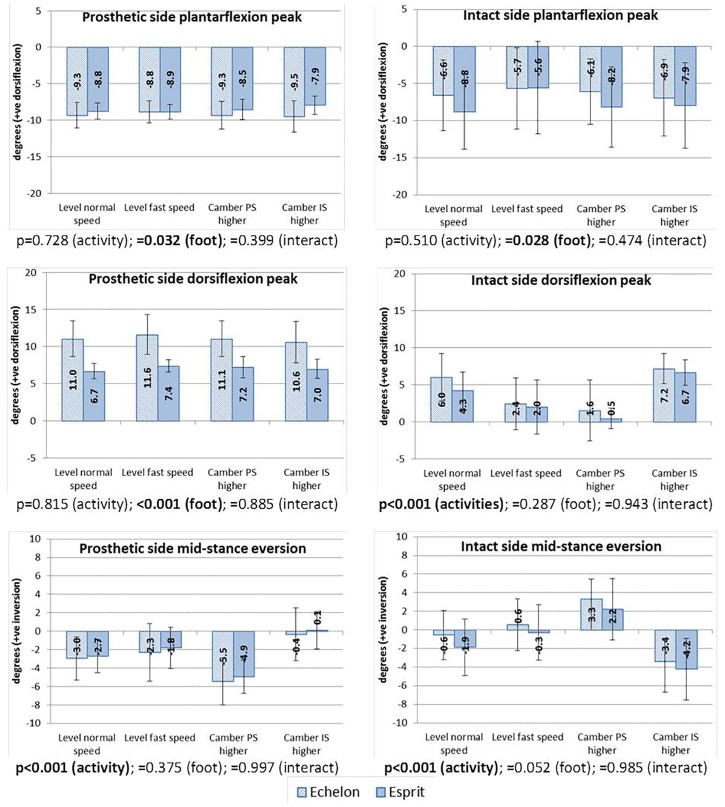
Mean values and standard deviations of the peak values in sagittal plane ankle angles and mid-stance ankle angles in frontal plane. The sagittal plane ankle movement in the Esprit foot is simulated by the deformation of the foot’s heel and forefoot keels. The p-values of the repeated measures two-way ANOVA are given below each chart. PS = prosthetic side, IS = intact side.

Fig 3 details the peak values in sagittal plane ankle angles during stance phase and the mid-stance values in frontal plane with relevant p-values of two-way repeated measurements ANOVA. In the sagittal plane, greater plantarflexion peak (p = 0.032) and more dorsiflexion peak during stance (p<0.001) were found at the prosthetic ankle when the Echelon foot was fitted. At the intact side, there was reduced plantarflexion when using the Echelon foot (p = 0.028). Differences caused by activities were found in the intact side maximum dorsiflexion, where the post-hoc results indicate that both level normal speed walking and camber walking with the intact side higher showed more dorsiflexion than level fast speed walking (comparing normal speed with fast speed: p = 0.023; comparing camber intact side higher with fast speed: p = 0.001) and camber walking with prosthetic side higher (comparing normal speed with camber prosthetic side higher: p = 0.002; comparing camber intact side higher with prosthetic side higher: p<0.001). In the frontal plane, significant differences were only found between the different walking conditions. The post-hoc results showed that when walking on the camber with prosthetic side higher, there was more eversion on the prosthetic side than the other walking conditions (comparing with level normal speed: p<0.001; comparing with level fast speed: p = 0.005; comparing with camber walking intact side higher: p<0.001) and more inversion on the intact side (p≤0.001 in all relevant post-hoc results). A converse change was found when walking in the other direction along the camber (p≤0.001 in all relevant post-doc results).

### Symmetry TSI and normalcy TSI of the ankle moment

The symmetry and normalcy TSI results are summarized in Table 2. Significant differences were only found between prostheses (foot) in both symmetry and normalcy TSI (p<0.001 in two TSI parameters) where the Echelon foot showed higher TSI values than the Esprit foot. Significant differences were not found between walking conditions (symmetry TSI: p = 0.119; normalcy TSI: p = 0.373) nor in interaction (symmetry TSI: p = 0.291; normalcy TSI: p = 0.417).

**Table 2:** Mean values (standard deviations) of the symmetry and normalcy TSI (%) of ankle moment.

Activities and foot models	Level normal speed		Level fast speed		Camber PS higher		Camber IS higher	
	Echelon	Esprit	Echelon	Esprit	Echelon	Esprit	Echelon	Esprit
Symmetry TSI	94.5(5.6)	91.9(5.0)	93.5(5.2)	90.9(4.4)	95.4(2.6)	89.5(6.0)	96.1(3.1)	93.0(5.0)
Normalcy TSI	97.8(0.3)	97.0(0.4)	97.9(0.5)	96.8(0.4)	97.8(0.5)	97.0(0.6)	97.9(0.2)	96.7(0.4)
Repeated measures two-way ANOVA results			Symmetry TSI: p = 0.119 (activity); **<0.001 (foot)**; = 0.291 (interact) Normalcy TSI: p = 0.373 (activity); **<0.001 (foot)**; = 0.417 (interact)					

PS = prosthetic side, IS = intact side.

### Questionnaire

Table 3 demonstrates the results of the short questionnaire that was answered by subjects after walking with each type of prosthetic foot. Overall in all walking conditions, subjects considered that the Echelon foot enables a more stable and safe feeling, while the Esprit foot was more difficult to swing, maintain balance, and give enough motion.

**Table 3:** Mean values (1: strongly disagree; 2: disagree; 3: neutral; 4: agree; 5: strongly agree) and standard deviations of the questionnaire results.

Activities and foot models	Level normal speed		Level fast speed^a^		Camber PS higher		Camber IS higher	
	Echelon	Esprit	Echelon	Esprit	Echelon	Esprit	Echelon	Esprit
Q1. This ankle makes me feel stable when I swing my sound leg.	4.8(0.4)	3.0(1.4)	4.5(1.0)	3.0(1.6)	4.6(0.5)	3.0(1.4)	4.4(0.5)	2.8(1.5)
Q2. This ankle makes my prosthesis hard to swing as I walk.	1.6(0.5)	3.2(1.8)	1.8(0.5)	2.7(1.5)	1.8(0.7)	2.6(1.1)	2.0(0.7)	2.4(0.9)
Q3. This ankle makes me feel off balance during walking.	1.6(0.9)	3.0(1.0)	1.5(1.0)	3.3(1.0)	1.4(0.5)	3.6(1.1)	1.4(0.5)	3.6(1.1)
Q4. This ankle does not provide enough motion and limit my movement.	1.6(0.9)	4.6(0.9)	1.5(1.0)	4.8(0.5)	1.8(0.8)	4.4(0.9)	1.4(0.5)	4.4(0.9)
Q5. Overall, this ankle makes me feel safe and confident during walking.	4.6(0.5)	2.2(0.8)	4.8(0.5)	2.3(1.0)	4.6(0.5)	2.2(0.8)	4.6(0.5)	2.2(0.8)

PS = prosthetic side, IS = intact side.

^a^ One subject rated “not applicable” in all fast speed walking questions.

### Data from control group

As some of the variables presented in this research are not commonly used in gait analysis, such as the frontal plane mid-stance ankle angle and TSI values of ankle moment, the data from the 12 subject NA group is provided in Table 4 for reference. However, due to the differences in gender and age between NA and TFA subjects, no comparison was made between the two groups.

**Table 4:** Mean values (standard deviations) of spatial–temporal parameters, the peak values in sagittal plane ankle angles and mid-stance ankle angles in frontal plane, the symmetry and normalcy TSI of ankle moment from non-amputee group.

Parameters	Level normal speed	Level fast speed	Camber left side higher	Camber right side higher
Walking speed (m/s)	1.12(0.10)	1.57(0.15)	1.19(0.11)	1.21(0.11)
Stride length (m)	1.30(0.07)	1.41(0.09)	1.29(0.07)	1.31(0.07)
Left step length (m)	0.64(0.04)	0.70(0.04)	0.64(0.04)	0.65(0.04)
Right step length (m)	0.65(0.03)	0.71(0.06)	0.65(0.04)	0.66(0.03)
Left stance time (%GC)	58.3(3.1)	57.7(1.5)	59.2(1.7)	58.6(1.8)
Right stance time (%GC)	58.9(1.4)	58.0(1.5)	59.3(1.7)	59.0(1.5)
Left maximum plantarflexion (degree)	-10.1(3.2)	-7.8(2.9)	-9.4(2.8)	-10.4(3.0)
Right maximum plantarflexion (degree)	-10.3(3.6)	-8.9(3.2)	-10.5(3.5)	-10.0(3.3)
Left maximum dorsiflexion (degree)	9.0(2.4)	7.7(2.6)	10.5(1.9)	8.0(1.5)
Right maximum dorsiflexion (degree)	8.2(2.6)	6.7(2.5)	7.5(2.7)	9.3(3.0)
Left mid-stance eversion (degree)	-5.2(2.1)	-4.5(2.0)	-7.6(2.4)	-1.7(2.4)
Right mid-stance eversion (degree)	-5.6(2.3)	4.9(2.5)	-2.5(2.1)	-8.6(1.8)
Symmetry TSI (%)	99.6(0.3)	99.7(0.4)	99.4(1.0)	99.6(0.5)
Normalcy TSI (%)	99.6(0.3)	99.2(0.7)	99.6(0.4)	99.4(0.5)

## Discussion

The primary aim of this research was to assess the kinematic and kinetic performance of a hydraulic prosthetic ankle/foot on level and camber walking conditions compared with a fixed prosthetic ankle/foot. This was carried out in two ways. Firstly, the kinematic performance in the sagittal was investigated on level ground walking at two speeds and on camber surfaces at normal speed. The second approach was to investigate if the hydraulic prosthetic ankle/foot device could provide better bio-mimicry of the resistance moment pattern compared with a fixed prosthetic device using symmetry and normalcy TSI as assessment tools. The secondary aim of this study was to assess the inversion/eversion movement of both types of prosthetic ankle/foot.

**Fig 4:**
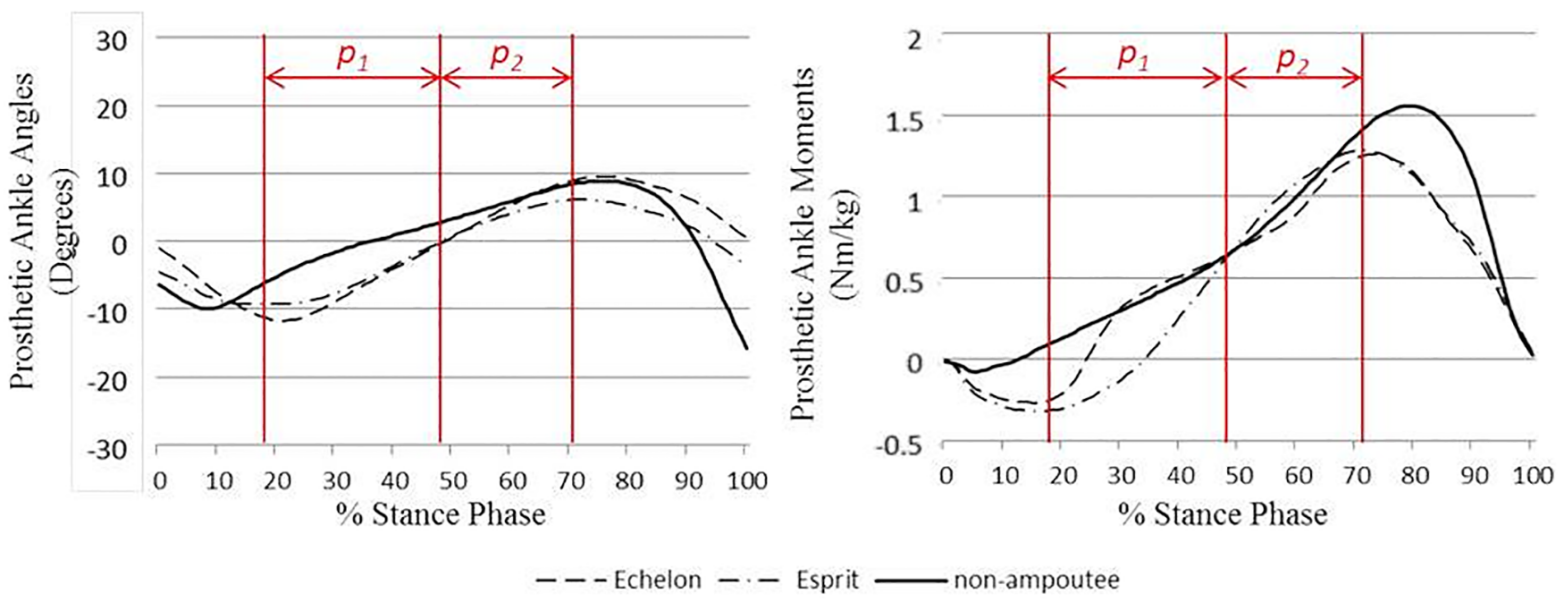
The mean values of the sagittal plane prosthetic ankle angles (left, +ve dorsiflexion) and prosthetic ankle moments (right, +ve plantarflexor) of subject (ID: TF4) during stance phase in level ground normal speed walking compared with the mean non-amputee group data. The sagittal plane ankle movement in the Esprit foot is simulated by the deformation of the foot’s heel and forefoot keels.

An improved prosthetic ankle moment, with better bio-fidelity, is considered to be a major advantage of hydraulic ankles. This is indicated by the significantly greater symmetry and normalcy TSI values compared with fixed ankle design (as showed in Table 2). Since similar patterns were found among subjects and with different walking conditions, the data from subject TF4 (Fig 4) in level ground normal speed walking is used for illustration. Based on the observation of ankle moment patterns from the two types of prosthetic ankle/foot, the changes that appeared at two periods in the gait cycle are believed to be the main contributor to the differences in TSI values. The first period (*p*_*1*_) is from maximum plantarflexion to the time that the prosthetic ankle rolls back to the neutral position (from about 18% of stance phase to 49% in subject TF4). In this first period (*p*_*1*_), the ankle moment of Echelon foot increased rapidly (from about 18% to 28% of stance phase) to a level similar to that of the non-amputees while the Esprit foot showed a more gradual rise in the plantarflexor moment. The second period (*p*_*2*_) is from the neutral position to the maximum dorsiflexion angle (from about 49% of stance phase to 72% in subject TF4). In this second period (*p*_*2*_), as with the non-amputees, the Echelon foot showed a concave curve, while that for the Esprit foot showed a more convex curve. According to Pitkin’s studies on the resistance moments in different types of prosthetic ankle/feet [15–17, 34], the fixed angle designs normally showed patterns similar to the Esprit foot, where the plantarflexor moment rapidly increases at the beginning of dorsiflexion and therefore a convex moment-angle pattern is obtained, which is the reverse of the biological ankles/feet as showed in non-amputees. Pitkin found that the traditional pattern in a fixed ankle produced a “stiff” feeling during ankle dorsiflexion in trans-tibial amputees (TTAs) [34]. Pitkin also calculated a substantial decrease in the stresses on the residuum of TTAs when a moment pattern that is closer to the biological ankle joint was applied to a mathematical model and hypothesised that the TFAs would receive similar benefits from biologically compliant prosthetic ankles and knees [16]. Therefore, the hydraulic ankle/foot could theoretically improve the walking comfort of TTAs and the higher symmetry and normalcy TSI of the resistance moment found with the hydraulic ankle/foot could explain the decreased peak internal stresses at the amputated limb that was reported in a previous study [4]. In TFAs, due to the influence of different prosthetic knees, future investigations with unified prostheses are required to link the bio-mimicking plantarflexor moment with socket comfort. In this research, one of the subjects (subject TF5) reported reduced comfort in the socket with the Esprit foot, which might further support Pitkin’s hypothesis with TFAs.

The benefits of hydraulic prosthetic ankles/feet were also found in the kinematics as the hydraulic ankle enabled an increased range of motion compared to the simulated movement provided by the Esprit in the sagittal plane during stance. Significantly greater values in the maximum plantarflexion (p = 0.032) and dorsiflexion (p<0.001) angles were found when the Echelon feet were used (as showed in Fig 3). The questionnaire results showed that subjects generally believed that the Esprit foot does not provide enough motion in contrast to their responses with the Echelon foot. This improvement also indicates that a hydraulic prosthetic ankle/foot could better adapt to steeper slopes than the fixed ankle designs. In a previous report that compared the same two types of prosthetic ankles/feet with TFAs, significantly increased self-selected level ground walking speed was noticed [2]. However, in this research, although the TFAs walked slightly faster, on average, with the Echelon foot in all walking conditions, it does not reach a statistically significant level. It should be noted that the comfortable walking speed of TFAs with hydraulic ankles/feet was 0.99±0.10 m/s and with fixed ankles/feet was 0.94±0.11 m/s in the previous study [2], while in this research, the TFAs were possibly more efficient and active walkers who walked at 1.13±0.19 m/s with Echelon foot and 1.10±0.13 m/s with Esprit (as showed in Fig 2), which are similar to the non-amputee group and showed limited scope for improvement. A significantly greater stride length was found with the Echelon foot (p = 0.026), which is considered to be caused by the slightly increased walking speed. However, although both prosthetic and intact sides showed consistently greater step lengths on average in all walking conditions, none of them reached a statistically significant level (p = 0.103 and 0.224 at the prosthetic and intact side respectively as showed in Fig 2). Since the stride length equals the sum of the step lengths on both sides, we conclude that the differences in a single step length do not allow the distribution of values on either side to reach a statistically significant level but a summation of the differences (from the addition of the step lengths) is large enough to show statistical significance in the stretched distribution. The intact side ankle kinematics was also found to be affected by the type of prosthetic foot devices, where the Echelon foot produced a reduced plantarflexion peak. However, author could not link this feature with either benefits or drawbacks in the amputee’s walking experience.

The significant differences in spatial–temporal parameters caused by increased walking speed on level walking were as expected, including the greater walking speed, stride length and step length (as showed in Fig 2) compared with normal speed. Some effects of camber ground walking were indicated by the difference in step length. Although there is significantly increased walking speed, compared with camber walking in both directions, the fast speed walking only showed significantly greater step length relative to the intact side when the prosthetic side is higher and at the prosthetic side when the intact side is higher. Also, for camber walking with the intact side higher, the intact side step length was found to be significantly greater than both level normal speed walking (p = 0.006) and camber walking from the other direction (p = 0.010). An explanation for these differences is unclear, but they may result from postural adjustments in order to supplement foot clearance for the higher foot during swing. The different activities also caused differences in ankle kinematics (Fig 3). A greater intact side maximum dorsiflexion was found in level normal speed walking and camber walking with the intact side higher compared with the fast speed walking and camber walking with intact side lower. This agrees with the trends seen in this data at the biological ankle joint in non-amputees (Table 4) in fast walking and for walking on camber ground. For the latter, relatively less dorsiflexion in stance of the lower foot will facilitate clearance of the higher foot. For the former, the data is supported by that found by Grant and Chester [35] and is probably due to an increase in plantarflexor power in stance as speed increases [36]. The changes in the frontal plane ankle movements on both sides were as expected in adapting to the ground condition and will be discussed later.

Heavier weight has been reported as a drawback of the articulating designs [37]. The Echelon foot was about 0.5 kg heavier than Esprit, which was hypothesised as being a main factor that might affect the swing of the prostheses because increased hip muscular effort has been reported with increased prosthetic shank mass [38] and the hip and knee moments during swing are increased with segment mass [20]. However, in contrast, the Esprit foot, which is lighter, gained a higher rating in the difficulty of swing in the questionnaire (Table 3). There are four possible reasons: (1) the TFAs that participated in this project are active prostheses users who use the prostheses on a daily basis and with comparatively strong residual limb and core strength are more capable of accepting heavier prosthesis weight; (2) the improved energy store and return in the hydraulic ankles/feet [1], to some extent compensate for the energy cost of the increased weight; (3) as shown in Fig 3, the neutral angle of the Esprit foot is greater in plantarflexion compared with the hydraulic device, which reduced the foot clearance during swing phase; and (4) the reduced comfort and feeling of instability during stance on the prosthetic side might affect the subject’s judgment of the swing phase. Previous work proved that increased prosthetic shank weight (no more than 100% of the intact side shank mass) would not significantly affect the TFAs’ walking speed and swing time [39], and the stride length and step length on the prosthetic side were increased by about 1 cm with slightly reduced walking speed (0.3 m/s slower) when 0.6 kg additional weight was added at the prosthetic ankle [39]. These observations are in generally agreement with the spatial-temporal parameters reported in this research.

Considering the designs of the two types of prosthetic ankle/foot, the side to side motion is mainly accomplished by the deformation of toe and heel springs and the ranges of changes between level walking and camber ambulation should be the same. This is demonstrated by the simulated mid-stance inversion/eversion angles as both Echelon and Esprit feet showed similar changes in the tendency and magnitudes during camber walking in both directions (p = 0.375 between prosthetic feet and p<0.001 between activities as showed in Fig 3). It was hypothesised that the inversion/eversion movement might be different between the prosthetic feet possibly because of changes in gait/posture and foot ‘roll-over characteristics’. But no difference between the feet was found in the results. The ranges of change in the inversion/eversion angles were also similar to the observations from non-amputees. However, in another study, it was reported that the inversion/eversion angle changes in prosthetic ankles were much smaller than a non-amputee group on a 6° camber walkway or 10% camber walkway [8, 9]. One reason could be that the camber used in this research is less steep (2.5°) so that the requirement of ankle adaption is reduced. Another reason could be the effects of different types and models of prostheses, however, the information of prosthetic devices was not given in the other studies. The ankle is the major adaptor to camber walking conditions as noticed from the data from the non-amputees (Table 4). Camber ambulation was hypothesised by this author as the walking condition that may most affect the ankle moment TSI due to the posture adjustment to adapt to ground condition. However, no statistically significant differences were found in either symmetry or normalcy between the different walking conditions the presented here. Again, the relatively small degree of camber might be an explanation. A future study with different degrees of camber walkway is needed to verify this assumption.

Both symmetry and normalcy TSI was computed in this research (Given in Table 2). A larger standard deviation was found in the symmetry TSI because one subject, subject TF1, showed about 10% lower TSI in all walking conditions compared with the mean value of the other four subjects. This is because this subject used intact ankle vaulting during mid-stance to support the prosthetic side foot clearance. This strategy produces one more plantarflexor moment peak at mid-stance in addition to the typical plantarflexor peak in late stance and causes reduced trend symmetry in the ankle moment pattern. Amputations always affect the intact side as well because the sound limb needs to provide adjustments or compensations in order to use prostheses and supplement the loss of function. Therefore the intact side ankle moment pattern of amputees also differs from non-amputees. This could be the reason that the mean symmetry TSI values of TFAs are lower than the normalcy TSI in all walking conditions. So it is suggested that the normalcy TSI should be used in the assessment of prosthetic ankles/feet during stance so that the influences from the individual intact limb adjustment could be reduced. The symmetry TSI is believed to be more applicable in the assessment of gait of an individual since asymmetry is a widely recognised characteristic of unilateral TFA gait and several complications of amputations were found to be linked with gait asymmetry [40, 41].

A main limitation in this research is that the marker set and modelling method is commonly applied to non-amputees. Because most of the mechanical structure of the prosthetic ankle/foot was covered by the foot shell and shoes, most foot markers cannot be placed in accordance with the mechanical structure. The detailed motion and deformation of the mechanical structure therefore were not recorded or observed during walking. Considering the inversion/eversion movement of the two models of prosthetic ankle/foot device is simulated by the differential flexion of the two halves of the split forefoot keel, the modelling approach used in this study provides limited information on this aspect. Some assumptions made in the conventional inverse dynamics approach for calculating internal joint moments were not entirely applicable to prosthetic segment even though the segment parameters were separately measured. Although some new modelling methods have been developed in the calculation of prosthetic joint power [42], to the authors’ knowledge, there is no published work that will permit improved calculation of prosthetic joint moments when assessing prosthetic ankles/feet that are similar to the ones used in this research. Another main limitation is that the ankle marker could not be placed at the same position on the two types of prosthetic foot due to the difference in the structure of the devices. Therefore a systematic bias was introduced to the results. The use of normalised ankle angle will have reduced the effects caused by the different ankle marker placements to some extent, but not completely. There are some other limitations. The test was of a non-blind design and this may affect the questionnaire results. The short questionnaire is not validated so it is just for providing further input when considering the analysis results. The subjects who participated in this research are good walkers that use their prostheses on a daily basis and they could adapt to a new prosthetic ankle/foot device in a relatively short fitting time. However a longer fitting period may well have affected the walking pattern of the subjects. A small number of subjects participated in this study which limits the capability of the statistical method (repeated measures ANOVA) applied and does not enable us to analyse for co-variates, e.g. duration of use of prosthetic device and subjects age, weight and height. Therefore the statistical results in this study are intended to provide indicative analysis only and further study is required to confirm our findings. For future studies, an increased number of TFA subjects and an extension to TTAs with monitoring of the socket stress is suggested. Involvement of other brands and models of hydraulic ankles/feet is needed to confirm if the improvements found with the Echelon foot could be considered as typical features of all hydraulic type ankles/feet. In the calculation of normalcy TSI, a reference group with gender and age matching subjects is suggested. And as previously mentioned, the camber walkway used in this research is of a relatively conservative dimension that was guided by the BSI Standards Publication “Design of buildings and their approaches to meet the needs of disabled people—Code of practice” (BS 8300:2009+A1:2010). However, based on the performance of the active prosthesis users in this research and the potential ground conditions that amputees may face outdoor, it is considered that a steeper camber might contribute to a better assessment of the prostheses.

## Conclusions

In conclusion, in all walking conditions (level ground normal speed walking, level ground fast speed walking, and camber walking from two directions), the hydraulic ankle/foot (Echelon) provides an increased range of motion in both plantarflexion and dorsiflexion during stance and better bio-mimicking of the ankle moment pattern than the fixed ankle design (Esprit). The inversion/eversion angle indicated that both types of the prosthetic ankles/feet allow adaption to a 2.5° camber from either direction. The questionnaire results suggest subjects are more satisfied with the hydraulic ankle/foot. Overall, bearing in mind the limitations outlined above, it is suggested that for active TFAs, the hydraulic ankle/foot enables an improved walking experience and comfort.

## Supporting information

S1 Dataset Time-normalised ankle angles (in frontal and sagittal planes) and moments (in sagittal plane) from five trans-femoral amputee subjects. A set of five trails from each subject in four walking conditions (level ground normal speed walking/level ground fast speed walking/camber walking with prosthetic side higher/camber walking with intact side higher) with two types of prosthetic ankle/foot (Echelon/Esprit). All data have been time-normalised to 101 points over stance phase and exported to excel files.
(ZIP)

S1 Questionnaire Questionnaire for subjective assessment of different prosthetic ankle/foot devices. (DOCX)
