# Supplementary material for: Kinematic and biomimetic assessment of a hydraulic ankle/foot in level ground and camber walking
Source: PLoS One. 2017 Jul 13;12(7):e0180836. doi: 10.1371/journal.pone.0180836 (PMC5509258; doi:10.1371/journal.pone.0180836)
Supplement: S1 Questionnaire — (DOCX) [file pone.0180836.s002.docx]

**
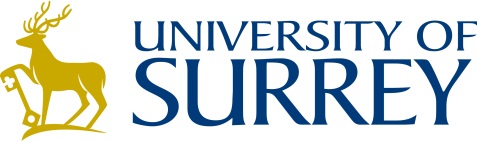
SUBJECT QUESTIONNAIRE**

**Title of Study:**

Comparison of Trans-femoral Amputees’ Prosthetic Gait when using Different Prosthetic Ankles/Feet

Participant ID Number: ________________________________________________________

Date of Attendance: __________________________________________________________

Name of Researcher: ________________________________________________________

Prosthetic Ankle/foot type: _____________________________________________________

**Instructions**

As you read each question, remember there is no right or wrong answer. Just think of YOUR OWN OPINION on the topic.

The rating scale from 1-5 is defined as follows:

1 Strongly Disagree

2 Disagree

3 Neutral (neither agree nor disagree)

4 Agree

5 Strongly Agree

NA Not applicable

**Example**

| 1. | It is important for me to have some milk every day. | 1 2 3 4 5 NA |
| --- | --- | --- |
| 2. | Over the past four weeks, the milk I had was delicious. | 1 2 3 4 5 NA |

This example shows that the person who answered these questions felt that having milk every day was not important at all to him. He also felt that the milk he had during the last four weeks tastes not too bad.

If he hadn’t drunk any milk in the last four weeks, he would have circled NA instead of circle a number.

**As in this example, circle a number that represents your opinion about the statement.**

During the tests, if you believe that there are issues that have not been covered by the questions, please write them down in the space provided on the final page. Any comments about this ankle (likes & dislikes) would be appreciated.

Please answer the following question according to your walking experience with the prosthetic ankle/foot you used in the laboratory. Please do NOT answer the questions until you have completed the relevant walking activities.

***1. Level ground walking (normal speed)***

| 1. | This ankle makes me feel stable when I swing my sound leg. | 1 2 3 4 5 NA |
| --- | --- | --- |
| 2. | This ankle makes my prosthesis hard to swing as I walk. | 1 2 3 4 5 NA |
| 3. | This ankle makes me feel off balance during walking. | 1 2 3 4 5 NA |
| 4. | This ankle does not provide enough motion and limit my movement. | 1 2 3 4 5 NA |
| 5. | Overall, this ankle makes me feel safe and confident during walking. | 1 2 3 4 5 NA |

***2. Level ground walking (fast speed)***

| 1. | This ankle makes me feel stable when I swing my sound leg. | 1 2 3 4 5 NA |
| --- | --- | --- |
| 2. | This ankle makes my prosthesis hard to swing as I walk. | 1 2 3 4 5 NA |
| 3. | This ankle makes me feel off balance during walking. | 1 2 3 4 5 NA |
| 4. | This ankle does not provide enough motion and limit my movement. | 1 2 3 4 5 NA |
| 5. | Overall, this ankle makes me feel safe and confident during walking. | 1 2 3 4 5 NA |

***3. Camber slope walking (prosthetic leg higher)***

| 1. | This ankle makes me feel stable when I swing my sound leg. | 1 2 3 4 5 NA |
| --- | --- | --- |
| 2. | This ankle makes my prosthesis hard to swing as I walk. | 1 2 3 4 5 NA |
| 3. | This ankle makes me feel off balance during walking. | 1 2 3 4 5 NA |
| 4. | This ankle does not provide enough motion and limit my movement. | 1 2 3 4 5 NA |
| 5. | Overall, this ankle makes me feel safe and confident during walking. | 1 2 3 4 5 NA |

***4. Camber slope walking (intact leg higher)***

| 1. | This ankle makes me feel stable when I swing my sound leg. | 1 2 3 4 5 NA |
| --- | --- | --- |
| 2. | This ankle makes my prosthesis hard to swing as I walk. | 1 2 3 4 5 NA |
| 3. | This ankle makes me feel off balance during walking. | 1 2 3 4 5 NA |
| 4. | This ankle does not provide enough motion and limit my movement. | 1 2 3 4 5 NA |
| 5. | Overall, this ankle makes me feel safe and confident during walking. | 1 2 3 4 5 NA |

***Notes:***

|  |
| --- |
